# CO_2_ mitigation or removal: The optimal uses of biomass in energy system decarbonization

**DOI:** 10.1016/j.isci.2021.102765

**Published:** 2021-06-24

**Authors:** Piera Patrizio, Mathilde Fajardy, Mai Bui, Niall Mac Dowell

**Affiliations:** 1 Centre for Environmental Policy, Imperial College, London, SW7 1NE, UK; 2 Centre for Process Systems Engineering, Imperial College London, London SW7 2AZ, UK; 3 Judge Business School, University of Cambridge, Cambridge, CB2 1AG, UK

**Keywords:** Energy resources, Energy policy, Energy sustainability, Energy systems, Energy flexibility

## Abstract

Owing to its versatility, biomass can be used for a range of CO_2_ mitigation and removal options. The recent adoption of end-of-century temperature targets at the global scale, along with mid-century economy-wide net zero emission targets in Europe, has boosted demand forecasts for this valuable resource. Given the limited nature of sustainable biomass supply, it is important to understand most efficient uses of biomass, both in terms of avoided CO_2_ emissions (*i.e*., substituted energy and economic services) and CO_2_ removal. Here, we quantify the mitigation and removal potential of key bio-based CO_2_ removal pathways for the transport, power, construction, and iron and steel sectors in Europe. By combining the carbon balance of these pathways with their economics, the optimal use of biomass in terms of CO_2_ avoidance and removal costs is quantified, and how these evolve with the decarbonization of the European energy system is discussed.

## Highlights

• Comparative assessment of the removal and avoidance value of bio-based pathways
• Biomass use for timber shows the highest CO_2_ removal potential
• Pyrolytic processes avoid 50% less emissions than BECCS to power routes
• The mitigation value of these pathways depends on the counterfactual scenario

## Graphical abstract

**Figure:**
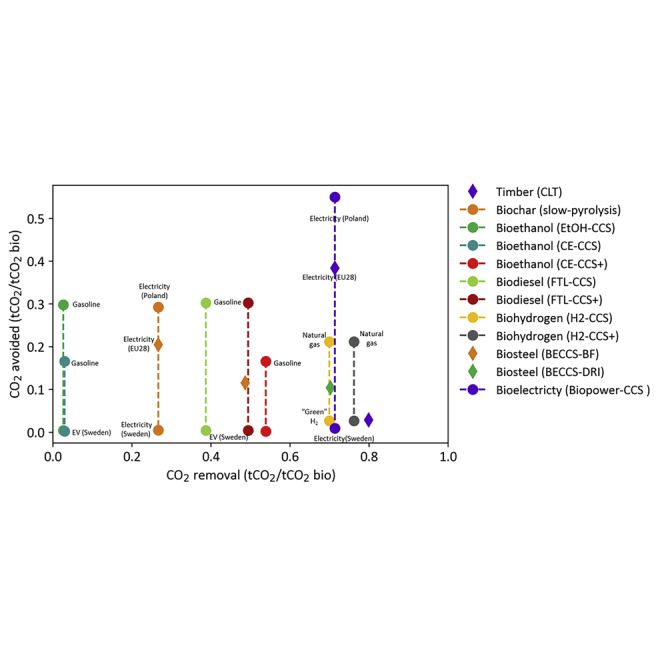

## Introduction

Global mitigation pathways capable of limiting the global temperature increase to 1.5°C above preindustrial levels require large amounts of biomass for use within the economy: the high energy demand scenario (SSP5) of sixth assessment report of the International Panel of Climate Change relies on up to 430 EJ of biomass use by 2100 (Huppmann et al., 2018; Rogelj et al., 2018). The reliance on large-scale bioenergy production for emission mitigation is expected to impact the amount of land available for food and the livelihoods of rural communities (Creutzig et al., 2013; Fajardy et al., 2019), as well as the delivery of other ecosystem services which may limit the deployment of biomass conversion pathways (Smith et al., 2015).

Biomass provides two main services for climate change mitigation. Its growth removes carbon dioxide from the atmosphere which can be stored for varying periods of time (carbon dioxide removal, or CDR). When managed and harvested in a sustainable way, biomass can also be used to avoid the release of carbon emissions to the atmosphere by directly replacing fossil fuel or by displacing high-carbon materials such as steel and cement (CO_2_ emission mitigation).

An inherent advantage of combining bioenergy with CO_2_ capture and storage (BECCS) relies on its potential integration within different conversion processes, such as combustion, gasification, and fermentation-based routes, thereby providing a wide range of low-carbon energy services, while providing long-term CO_2_ removal. In integrated assessment models (IAMs), the main BECCS pathways represented are typically biomass conversion to electricity in large-scale combustion plants and biomass conversion to liquid fuels. Bioelectricity with carbon capture and storage (CCS) can be valuable to decarbonize the power sector as a source of low carbon dispatchable power (Daggash and Mac Dowell, 2019; Truong et al., 2019; Heuberger et al., 2020). At the global level, however, a recent study shows that the deployment of bioelectricity-CCS plants is mainly driven by the demand for CO_2_ removal, and would still get deployed without providing decarbonized electricity (Fajardy et al., 2020). This suggests that, in the context of stringent CO_2_ emissions limitations, BECCS plants may have a greater CO_2_ removal value than mitigation value.

The production of liquid fuels via BECCS (*i.e.*, biofuels) can provide a substantial contribution to transport decarbonization in the mid-century stabilization scenarios (Muratori et al., 2017; Muri, 2018). Hydrogen can be used to decarbonize multiple sectors at a national level, *e.g*., industry, transport, and heating. Assuming high capture rates of CO_2_ (*i.e.*, greater than 90%), biomass for hydrogen production is presented as an energy efficient biomass utilization route to CO_2_ removal (Energy Systems Catapult, 2020). However, CO_2_ removal costs of biohydrogen are generally high due the high capital cost associated with its production (Bui et al., 2020). The potential integration of biomass and/or CCS in hard-to-abate fossil-intensive industries such as cement, chemicals, and iron and steel is also gaining increasing attention in the scientific literature (Mandova et al., 2019; Tanzer et al., 2020). In addition, countries such as the UK, Sweden, and Norway are currently proposing low-carbon roadmaps featuring the deployment of large-scale CCS infrastructure to support the decarbonization of key industrial clusters (Committee on Climate Change, 2019; Global CCS Institute (GCI), 2020; Karlsson et al., 2020).

While there is an abundance of evidence on the value of different biomass conversion routes within the energy systems (Codina Gironès et al., 2017; Mandova et al., 2018; Mesfun et al., 2018; Korberg et al., 2021), there is a paucity of evidence in the context of the mitigation services that these routes can provide. As highlighted in a recent review on carbon accounting studies (Tanzer and Ramirez, 2019), there is a lack of clarity around the quantification of the net CO_2_ captured, used, avoided, and removed within each biomass mitigation pathway. The amount of CO_2_ captured usually refers to the CO_2_ captured at the process level and can be significantly higher than the net amount of CO_2_ removed from the atmosphere, which accounts for life cycle fossil CO_2_ emissions (Smith and Torn, 2013; Fajardy and Mac Dowell, 2020). This is particularly important when comparing costs, which may differ greatly between $ per ton of CO_2_ captured and $ of per ton of CO_2_ removed (Daggash and Fajardy, 2019). Similarly, the amount of CO_2_ avoided is very much dependent on the counterfactual considered (Committee on Climate Change, 2018). Therefore, CO_2_ avoided potential, which is a relative value, should not be conflated with CO_2_ removal potential, which is an absolute value.

**Figure 1:**
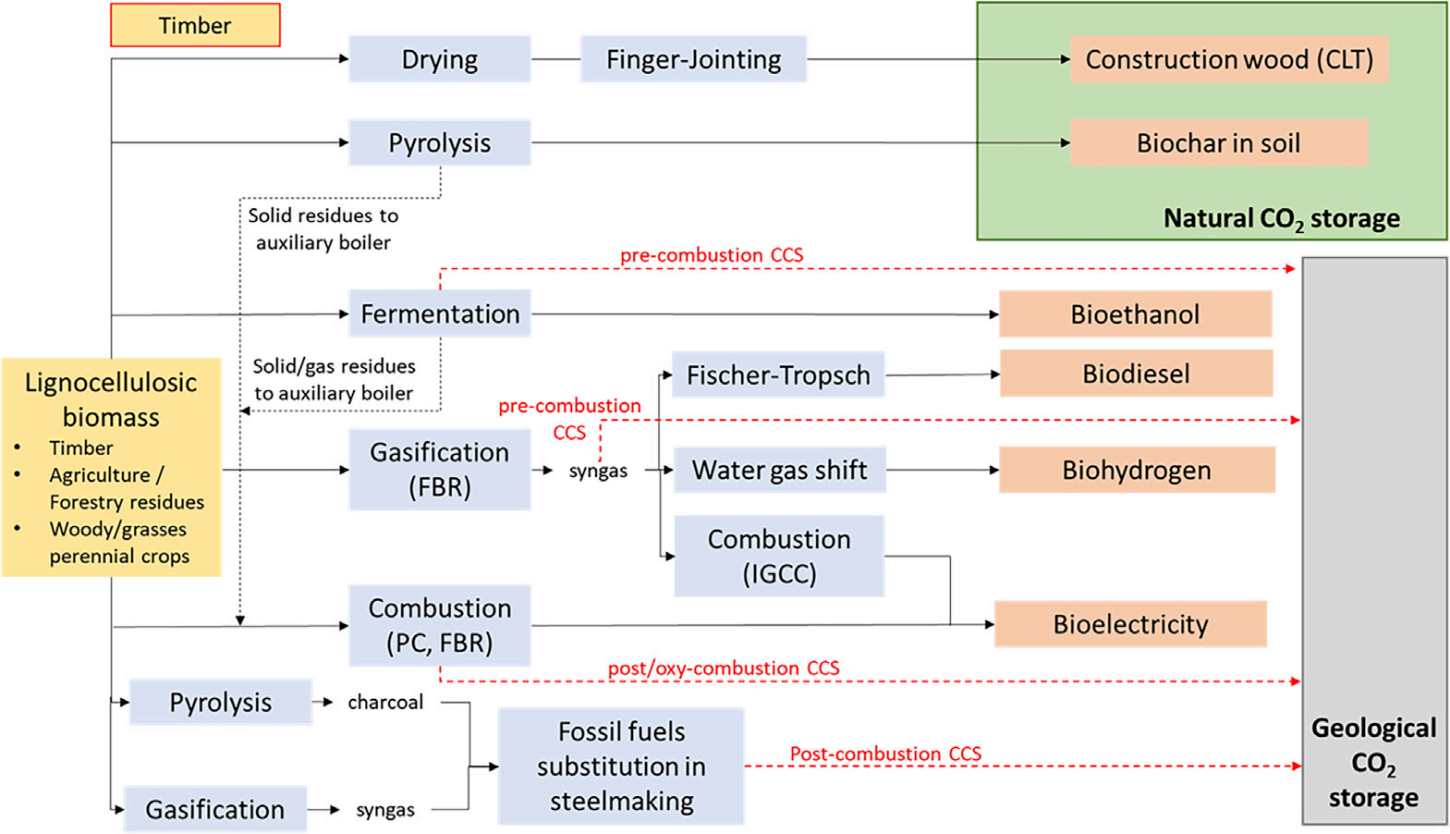
Biomass-based CO_2_ removal pathways considered in this study A detailed process characterization of each pathway is reported in the supplemental information (SI).

The relative mitigation potential of each pathway is also the result of biophysical factors (*e.g.,* biomass characteristics), process specifications (*e.g*., energy efficiency), regional variations (*e.g.*, land availability, yield), and the counterfactual scenario considered, among others (Fuss et al., 2018). Understanding the best uses of this scarce resource as a function of the regional context and of the pace of the energy system decarbonization is, therefore, key. This study aims to provide a transparent framework to quantify the carbon balance of selected biomass-based CO_2_ removal pathways for the power, transport, and industrial sectors in Europe (Figure 1). It should be noted that the adoption of biomass could also represent a valid mitigation option for the European residential sectors since most heating systems are currently fossil based. However, considering the requirement for achieving 85%–95% emission reduction in the residential sector by 2050 (Knobloch et al., 2019), the adoption of heat pumps for space heating is often seen as the most promising technological option (Thomaβen et al., 2021), provided the availability of zero-carbon electricity.

Here, biomass conversion routes for CO_2_ removal are categorized into two groups: pathways which lead to geological storage of CO_2_ and pathways which rely on CO_2_ storage in natural sinks. Geological CO_2_ storage pathways involve the capture, transport, and storage of the CO_2_ released upon conversion of the biomass to provide some form of energy service. These pathways, labeled as BECCS, are associated with the production of biofuels, bioelectricity, and bioheat or the adoption of biomass in industrial processes, such as steelmaking.

The BECCS pathways predominant in IAMs involve biomass combustion such as a pulverized combustion boiler (PC) to produce electricity. Alternatively, biomass can be adopted in fuel production routes, involving gasification and Fischer-Tropsch (FT) synthesis or gasification with steam reforming of the syngas and water gas shift. Grains (*i.e.*, first generation) and cellulosic biomass (*e.g.,* perennial grasses, woody biomass, agricultural residues) can also be converted to ethanol through fermentation. After fermentation, lignin residuals can be combusted in an auxiliary boiler to generate electricity (Humbird et al., 2011). As for the pathways relying on natural CO_2_ sinks, this study considers (i) the sequestration of biogenic carbon in soils *via* the pyrolytic production of biochar and its application to field and (ii) the production of cross-laminated timber (CLT), a widely used engineered wood for the substitution of reinforced concrete in the construction sector. A detail process characterization of each pathway depicted in Figure 1 is available in the supplemental information (SI).

## Results

For each of the conversion routes presented in Figure 1, our analysis considers the main costs occurring at various stages of the biomass value chain as detailed in Table S2. The net CO_2_ removal associated with the proposed biomass pathways is calculated as the physical amount of biogenic CO_2_ fixed in geological or natural storage to which the life cycle fossil emissions of this pathway (*e.g*., biomass production) are subtracted. The amount of CO_2_ avoided is somewhat more complex to quantify as it is entirely dependent on the counterfactual, chosen for each scenario. When possible, high, average, and low carbon intensity counterfactuals were chosen to determine a CO_2_ avoidance range. Hence, in the case of the production of bioelectricity, we considered the carbon intensity of power grids in Poland (high) and Sweden (low) while the average carbon intensity of European electricity has been adopted as the medium scenario.

The production processes associated with some of these pathways, *i.e.*, when biomass is converted into biofuels or adopted in steelmaking processes, are characterized by the presence of multiple CO_2_ outlet streams. Thus, for biofuel production routes, we distinguish between a base case approach where CO_2_ is captured only from the high concentration stream and an alternative configuration (CCS+) considering process modifications that enable higher CO_2_ capture rates. In iron and steel plants, biomass can partially substitute pulverized coal or natural gas as fuel for the iron making processes. Hence, for these routes, we considered only the capture of CO2 from the blast furnace (BF) or direct reduction of iron (DRI) flue gases, which are the CO_2_ streams containing biogenic carbon. Background data for the computation of the carbon removed and avoided within each pathway are reported in the SI (Tables S3 and S4).

### The optimal uses of biomass pathways

**Figure 2:**
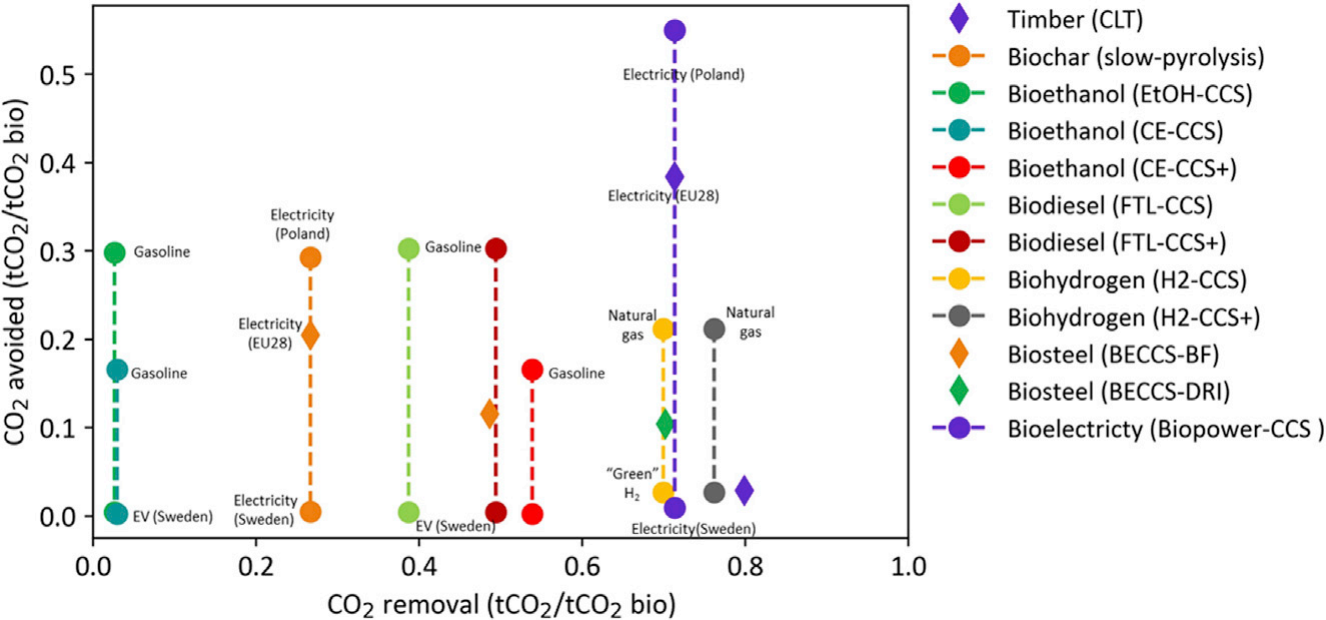
CO_2_ removal and CO_2_ avoided for a range of bio-based CO_2_ removal pathways and counterfactual scenarios Pathways associated with the production of biomaterials, i.e., CLT bio-steel, are compared to traditional production process, i.e. concrete in construction and steel from BF and DRI production routes. For pathways involving the production of bioelectricity, grid electricity in Poland and Sweden has been adopted as high and low counterfactual scenarios

As shown in Figure 2, the optimal use of biomass depends largely on the service for which it is deployed: mitigation or removal. Biomass use for timber in replacement of concrete shows the highest CO_2_ removal potential. This is because after the conversion of roundwood into dimensional timber, the adoption of the wood products for construction requires minimal additional processing. However, this pathway also exhibits a mitigation potential of 0.05 tCO_2_ t_dm_^−1^ due to the low substitution rates available for this product. In fact, in accounting for the carbon avoided by timber building structures, a common practice is to refer to a benchmark building unit, dimensioned to meet the same structural characterization. The weight substitution factor of cement with wood for these structures is lower than 30% since timber cannot replace some of the functional properties of cement, in addition to having a lower volumetric density. Thus, when considering a benchmark building structure, the mitigation potential of replacing one ton of cement with timber is lower. Among the biofuel pathways, the higher capture rate of biodiesel plants compared to bioethanol plants results in biodiesel-CCS having a CO_2_ removal potential five times higher than that of bioethanol-CCS. Only when the capture rate is maximized, as for the case of CE-CCS + route, the adoption of biomass for bioethanol production provides a valuable removal service. BECCS for the steel industry offers a lower removal value when used in (BF routes compared to DRI, as the biomass needs to be converted into charcoal before being integrated into the BF, which involves a production process with a conversion efficiency of 63% (Tanzer et al., 2020). Similarly, given the high capture rates obtained in gasification routes, adopting biomass for H_2_ production can remove up to 1.3 tCO_2_ t_dm_^−1^, corresponding to 0.76 tCO_2_ tCO_2bio_^−1^ when miscanthus is adopted as feedstock.

**Figure 3:**
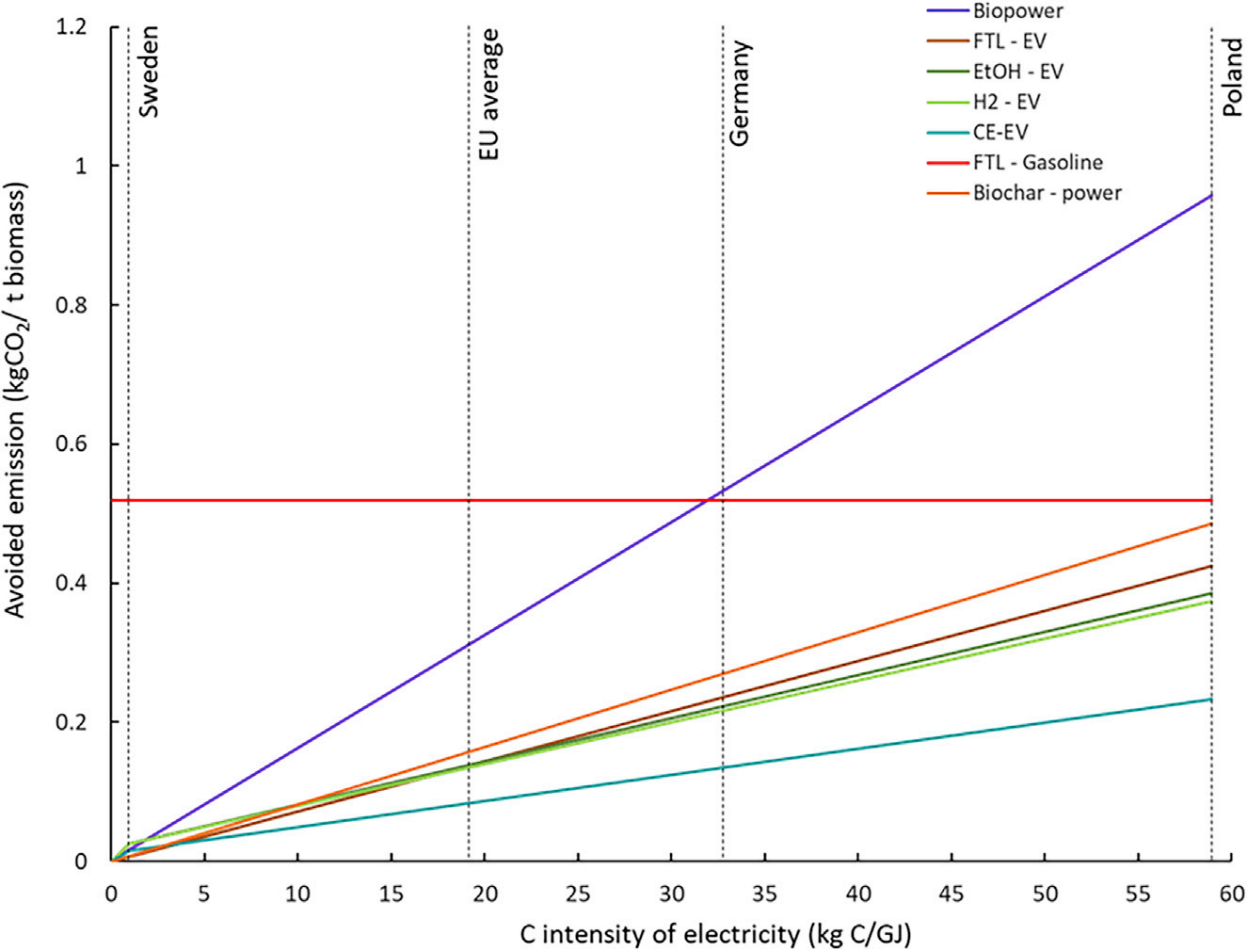
Mitigation potential of selected biomass pathways as a function of carbon intensity of the power grid The vertical dashed lines at 1, 18.8, and 59 kg C GJ^−1^ denote the carbon intensity of Sweden, EU28, and Poland electricity generation mix in 2016, respectively, according to the European Environmental Agency (EEA) database.

The mitigation impact of each pathway depends on the carbon intensity (*i.e.*, the mass of carbon emitted per unit of total energy produced) of the counterfactual energy product and the product/fuel substitution factor. As shown in Figure 3, adopting bioelectricity-CCS routes in countries with highly carbon intensive power grids, such as Poland, offers a mitigation potential of 0.96 tCO_2_ t_dm_^−1^._._ As expected, the cumulative avoided emissions for BECCS to electricity are essentially zero when the carbon intensity of the electricity mix is also zero. Hence, in low-carbon power grids, such as those in the Nordic countries, biomass would provide a much greater value in decarbonizing the transport sector, particularly if FT production routes are adopted, as the ratio of fuel over electricity production is maximized.

Because electric vehicles (EVs) have higher energy conversion efficiency than conventional gasoline and diesel cars, biomass-derived fuels exhibit an energy substitution factor as low as 26% when EVs are considered as the counterfactual scenario. Hence, in countries where EVs are available, adopting BECCS for biofuel production have little mitigation value, even when considering a highly carbon intensive energy generation mix. To calculate CO_2_ mitigation potential of biochar, both the co-production of bioelectricity and the reduction in fertilizer-induced N_2_O emissions were considered. As Figure 3 illustrates, biochar CO_2_ mitigation potential is mainly driven by the carbon intensity of the electricity grid, while the reduction in fertilizer use and related emissions play a secondary role. It is important to note that additional environmental benefits may also arise from the adoption of biochar for soil enhancement, such as increased crop yield, which could lead to higher CO_2_ avoided in the long term (Woolf et al., 2010).

### The value of biomass pathways in energy system transition

**Figure 4:**
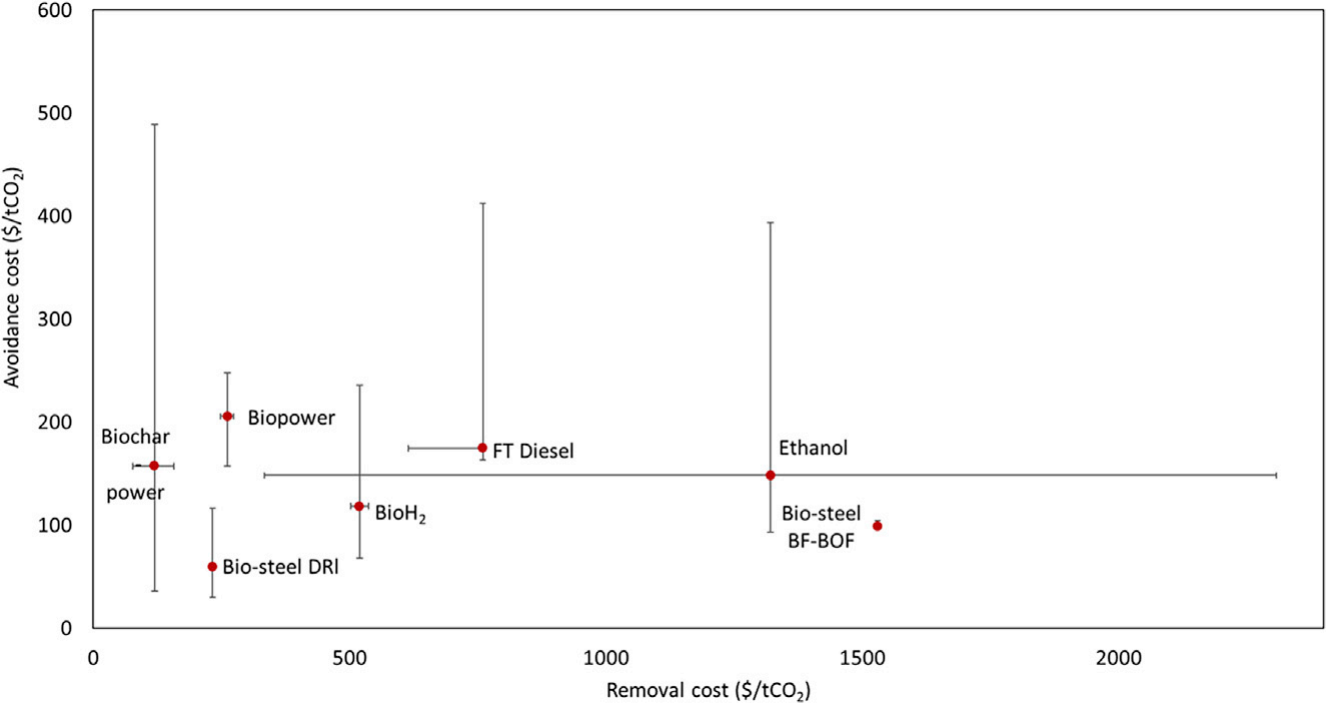
CO_2_ removal and CO_2_ avoidance cost for the selected biomass-based products The variability of avoidance costs shown reflect the counterfactual scenarios used in this study. Removal cost variation is associated with the range of CO_2_ capture rates available for each pathway and with the type of feedstock adopted in slow pyrolysis processes. For the analysis, we assigned a cost of $ 30 tCO_2_^−1^ for transport and storage in geological sinks (i.e. BECCS for power, iron and steel routes ,and biofuels with CCS). The timber production route, displaying a removal costs of 2110 $/t CO_2_, has been omitted from the chart as an outlier to allow focus on other technologies. Circles represent average CO_2_ avoidance and removal cost values.

Figure 4 combines the conclusions obtained from the carbon balance presented above with the techno-economic analysis (Tables S1 and S2) to derive the value of biomass-based pathways over time. Since the mitigation value of biomass is higher when the energy systems are yet to be decarbonized, pathways with lower avoidance cost are preferred in the short-medium term. Conversely after 2030, when high CO_2_ removal rates are to be realized by removing carbon from the atmosphere, the service provided by biomass as a CDR option is vital. The large variation in removal cost observed for ethanol is mainly associated with the low carbon removal potential of its production process, *i.e.*, the low share of biomass carbon (14–21%) that can be separated during the fermentation stage. Nevertheless, because of its lower capital cost compared to other biofuel routes, the production of ethanol exhibits a high mitigation value in the short term and can contribute to the decarbonization of fossil fuel-based transport sector at an average CO_2_ avoidance cost of 170 $ tCO_2_^−1^.

The lower mitigation potential of biochar power (*i.e.*, slow pyrolysis) compared to biopower CCS to offset fossil electricity is observable in Figure 4. This is reflected by CO_2_ avoidance costs ranging from $66 to 490 tCO_2_^−1^, with the upper value being associated with the offset of electricity from a low carbon energy generation mix (Sweden). This notwithstanding, bioelectricity can be produced at a lower cost in pyrolysis plants compared to large-scale biomass combustion plant with CCS; when considering miscanthus as biomass feedstock, this corresponds to $ 73 t_dm_^−1^ and $230 t_dm_^−1^, respectively. Hence, despite its lower removal potential, slow pyrolysis can remove carbon at a cost of 77–125 $ tCO_2_^−1^, depending on the feedstock adopted in the production process. Finally, DRI steel production routes (*i.e.*, BECCS-DRI) represent the best biomass utilization pathway owing to the high biomass utilization share, which lower the overall removal cost, and the high carbon intensity of the fuel being offset (*i.e.*, natural gas). However, it should be noted that most of the steel production across Europe is realized *via Blast Furnace-Basic Oxygen Furnace* (BF-BOF) production routes. Hence, the steelmaking industry could integrate biomass-derived charcoal in BF as a short-term solution, while progressing toward more sustainable ways of producing steel after 2030.

## Discussion

According to IAMs, efforts to keeps global temperatures “well below” 2°C will imply large-scale biomass utilization by 2050, mostly via BECCS deployment. Several studies (Smith et al., 2015; Anderson and Peters, 2016; Heck et al., 2018) have warned that the reliance on a single or restricted portfolio of CDR technologies to reach these targets not only triggers potential irreversible ecosystem impacts (*e.g.*, through land use change, water use) but also hinders the simultaneous implementation of other biomass energy strategies (Woolf et al., 2010). Considering the full portfolio of bio-based CO_2_ removal pathways in IAMs is, therefore, essential to maximize the removal of carbon emissions and to address unavoidable resource competition among them.

This study provides a comparative assessment of the value of a range of bio-based CO_2_ removal pathways in terms of CO_2_ emission avoidance and CO_2_ removal, and it also highlights the role of the counterfactual scenario when assessing the mitigation potential of a technology. Overall, a large-scale biomass combustion plant with CCS, though one of the lowest ranking pathways in terms of energy efficiency, provides the greatest CO_2_ mitigation potential in a carbon intensive grid such as Poland. When considering the same counterfactual scenario, the production of bioelectricity in pyrolytic processes avoids around half of the emissions avoided in BECCS to power routes since slow pyrolysis processes tend to maximize the biochar-to-electricity output ratio.

In addition, since the mitigation service of biomass is more valuable in fossil-based energy systems, bioethanol represents a cheap low-carbon alternative to gasoline due to its low production cost. In the longer term, *i.e.* after 2030, when increasingly higher rates of CO_2_ emissions are expected to be removed from the atmosphere, the transport sector could opt for pathways involving higher CO_2_ captures rates, such as FT diesel or bio-hydrogen production pathways. However, it is important to note that all BECCSs to fuel pathways investigated here exhibit a very modest mitigation potential when compared to EVs, especially when low-carbon electricity is available. In contrast to many other pathways, biomass use in BF-BOF steelmaking could be facilitated with modest investment costs. While our results confirm that higher carbon removal can be achieved when biomass is adopted in direct reduction steelmaking with electric arc furnace (DRI-EAF), the shift from current steel production technologies to alternative technologies, such as top gas recycling or DRI with CCS, requires major investments and should be regarded as the most effective in the long term (Suopajärvi et al., 2018).

Some of the biomass conversion routes presented in this study are characterized by significant techno-economic barriers which currently hinder their large-scale implementation. In bioethanol production routes, lignocellulosic feedstocks require several steps such as pre-treatment and enzymatic hydrolysis processes to allow the breakdown of sugars during fermentation. These processes make up of around 60% of the production cost and represent the greatest barrier for the commercialization of cellulosic bioethanol (Wyman, 2007). In addition, there is currently not one single process that is suitable and optimized for all biomass type, which challenges the deployment of this technology at a large scale. Similarly, the lack of publicly available guidelines on how to produce standardize biochar with reproducible characteristics, together with the uncertainties associated with its agronomic value, might hinder the adoption of slow pyrolysis processes at scale. Before farmers are likely to take up the use of biochar, it is probably necessary for the positive (and any negative) effects of biochar addition to be properly understood (Shackley et al., 2011). Finally, while bioenergy and CCS are established concepts in the context of steel production, at the time of writing, the use of BECCS in the steel industry has yet to be demonstrated in practice. Research and development initiatives under the “CO_2_ Breakthrough Program” have been investigating the potential for developing breakthrough technologies that hold the promise of large reduction in CO_2_ emission in the iron and steel industry (Fischedick et al., 2014). Among the technologies with the highest long-term potential, the direct reduction of hydrogen (H_2_-DR) with electric arc furnace has been proposed as an alternative mitigation route to BECCS in the iron and steel industry. However, for H_2_-DR routes to be a low-carbon option, low operating costs need to be achieved, which is only possible if abundant and cheap electricity is available.

### Limitation of the study

This study provides a comparative assessment of the value of a range of bio-based CO_2_ removal pathways in terms of CO_2_ emission avoidance and CO_2_ removal and costs. Other pathways relying on natural sinks might offer a higher technology readiness level as opposed to pathways reliant on geological storage. However, determining their overall removal and mitigation potentials is complicated by issues related to the permanence of storage and the uncertainty around potential co-benefits. In addition, while the permanence of CO_2_ storage in geological sinks has been highly discussed and recent research suggests that leakage rates are negligible (Miocic et al., 2019), in the case of natural CO_2_ sinks, there is a higher risk of CO_2_ being re-emitted back into the atmosphere in a shorter time frame. This would result in a lower amount of net removal in the case of timber wood and biochar.

In conducting the analysis of biochar production pathways, it is also assumed that 68% of the carbon in the fresh biochar remains stabilized in the long term. This is a simplification since the long-term stability of recalcitrant carbon will vary depending upon the feedstock, the pyrolytic production conditions, and the receiving soil. However, at present, there is no reliable method for calculating the long-term stability of biochar; hence, a common value found in literature has been used (Hammond et al., 2011; Shackley et al., 2011).

Finally, a limitation of this study that applies to all pathways includes the assumption on the availability of low cost and low carbon intensity biomass. As the scale of the biomass potential is highly uncertain once accounting for economic, social, and environmental impacts, real-world implementation of biomass in the energy and industrial sectors requires consideration of all these factors at the local and regional levels (Buck, 2018; Patrizio et al., 2018). In this context, accounting for the regional ecosystems' impacts such as the land and water footprint associated with crop cultivation and use within each conversion pathway is key. This particularly applies to pathways with low biomass conversion efficiencies such as slow pyrolysis processes, where the removal of one ton of carbon is associated with high land and water requirements.

## STAR★Methods

### Key resources table

**Table:** 

REAGENT or RESOURCE	SOURCE	IDENTIFIER
**Deposited data**		

Capex and Opex of biomass conversion processes	This paper (supplemental information, Table S2)	
Process efficiencies and main assumptions of biomass conversion pathways	This paper (supplemental information, Table S3)	
Fuel emissions and costs	This paper (supplemental information, Table S5)	

### Resource availability

#### Lead contact

Further information and requests for resources should be directed to and will be fulfilled by the lead contact, Dr. Piera Patrizio (p.patrizio@imperial.ac.uk).

#### Materials availability

Not applicable.

#### Data and code availability

• The attached supplemental information file includes all dataset generated or analyzed during this study
• This paper does not report original code
• Any additional information is available from the lead contact upon request

### Method details

#### Selected biomass-based CO_2_ removal pathways: process performance and costs

##### Biomass for construction wood (CLT)

Timber is currently used in construction, along with steel and reinforced concrete. Aside from dimensional sawn timber, softwoods are also processed into structurally optimized building materials known as ‘engineered timber’, the benefits of these wood composites, include increased dimensional stability, more homogeneous mechanical properties and greater durability. Cross-laminated timber (CLT) has strength parallel to that of reinforced concrete, however, timber cannot match modern high-strength concrete in compression (Ramage et al., 2017). Of all the manufacturing processes associated with converting roundwood into dimensional timber, kiln drying of softwood accounts for up to 92% of total manufacturing energy. The use of wood as a structural material often has the consequence of introducing other materials to achieve certain performance requirements; concrete is often used to achieve acceptable floor vibration or to achieve thermal mass (Ramage et al., 2017). Hence, in accounting the carbon avoided by CLT building structures, a common practice is to refer to a benchmark building unit (BU), dimensioned to meet the same loading condition and having the same footprint areas and building heights (Skullestad et al., 2016). The substitution factor (Sub) is then calculated considering the density (p) and volume (V) utilization of the timber structure compared to a concrete-based one.

$$Sub_{timber}=\%V_{timber}*\frac{p_{timber}}{p_{concrete}}\;$$

Across the UK, an increasing number of non-residential buildings are being built using engineered sawn wood products (BioComposites Center, 2019) such as cross-laminated timber (CLT) and glue laminated timber (glulam). CLT production facilities in Europe report an average production capacity of 25,500 m^3^ y^−1^, corresponding to 12.68 kt y^−1^ of biomass. Production prices are reported between $600–742 m^−3^, the large variation depending on assets utilization. A recent techno-economic study analyzed the impact of capacity factor and found that the cost of CLT increased by 33% for facilities reducing their operating capacity to 50% (Brandt et al., 2019). In this study, we adopted the techno-economic findings from Brandt et al. (2019) and considered a reference plant size of 52,000 m^3^ y^−1^ CLT, operating at 80% capacity.

##### Biochar for soil carbon sequestration (slow pyrolysis)

Biochar is a carbon rich material produced during biomass pyrolysis. Biogenic waste materials suitable for biochar production include crop residues, food and forestry wastes, and animal manures. Biochar's climate-mitigation potential stems primarily from its highly recalcitrant nature (Woolf et al., 2010), which slows the rate at which photosynthetically fixed carbon (C) is returned to the atmosphere. In addition, the production of biochar potentially yields several co-benefits including the production of bioelectricity, thus generating avoided emissions as a function of the carbon intensity of the displaced electricity. The amount of biochar and bioelectricity generated depends on the operation of the process. If used as bioenergy to offset fossil-fuel emissions, biochar has an offset ratio between 0.8 and 0.92 when the process is optimized for biochar production or for electricity respectively (Gaunt and Lehmann, 2008). Studies also suggest that biochar application in soil can improve soil quality (Gaunt and Lehmann, 2008; Biederman and Stanley Harpole, 2013; Hagemann et al., 2017), potentially increasing net primary productivity and thereby reducing economic pressure to convert native lands to agricultural production, or land use change. Soil biochar application may also directly reduce GHG emissions from soil, by reducing both the need for nitrogen fertilizer, and the subsequent N_2_O emissions from the soil by unit of applied fertilizer. About half of the C fixed in the biomass returns to the atmosphere during the process (Woolf et al., 2010). The C fraction of biochar can fluctuate between 25% and 70% mainly depending on the ash content (and, to a lesser extent, H and O content) of the feedstock, with wood waste and animal manure on the upper and lower bound of this range, respectively. Biochar produced from residues of crops and grasses is generally more degradable than that from wood, which is attributed to inert properties of various feedstocks, such as the high lignin content. A recent study (Wang et al., 2016) using 128 observations of biochar-derived CO_2_ from 24 studies found biochar-to-soil had a mean residence time (MRT) of 107 years, confirming its ability to store carbon over a long period of time. Various pyrolysis technologies yield different proportions of biochar and syngas, which is typically used to generate electricity. In general, pyrolysis maximizes biochar production at temperatures between 300 and 700°C (slow pyrolysis) and maximizes condensable vapors production, *i.e.* bio-oil, at higher process temperature (fast pyrolysis). Technological development of fast pyrolysis is more advanced than slow pyrolysis, with several medium scale facilities having been constructed over the last decade to produce bio-oils (Brown et al., 2015). Accurate capital and operational cost estimates of slow pyrolysis facilities are scarce, given the commercial immaturity of this technology. A comprehensive literature review on biochar production technologies (Lehmann and Joseph, 2012) reported production costs in the range of $100–1500 ton biochar^−1^ for biomass costs of $47.4–82.5 dry ton^−1^. The wide variation in cost can be attributed to the different size/scales and process conditions available in literature and most importantly, to the inclusion of potential revenue streams in the assessment, *e.g.*, gate fees, co-production of electricity and bioliquids. Shackley et al. provided a detailed cost benefit analysis of biochar production in the UK, considering three potential unit sizes and potential economic-benefits such as avoided gate fees, sales of electricity and the revenues from renewables obligation certificates (Shackley et al., 2011). The work adopted data from one of the few demonstration units for slow pyrolysis to derive operational costs and estimate the value of electricity generated; this data was used to form the basis of the economic analysis in this study. Data from gasification plants were used to inform the calculation of capital expenditure, ranging between 0.9 and 41.25 M$ for the selected plant scale (Shackley et al., 2011).

##### BECCS for bioethanol (EtOH-CCS, CE-CCS and CE-CCS+)

Grains (*i.e.*, first-generation) and cellulosic biomass (*e.g.,* perennial grasses, woody biomass, agricultural residues) can be converted to ethanol through fermentation with an energy efficiency between 29 and 44% [All energy efficiencies are in percentage lower heating value (LHV)] (Humbird et al., 2011; IEAGHG, 2014). As CO_2_ is already separated during the fermentation stage, conversion efficiencies already include the energy penalty associated with CO_2_ capture. Compared to first generation feedstocks, lignocellulosic biomass requires additional process steps to produce ethanol, *i.e.* biomass pre-treatment followed by enzymatic or acidic hydrolysis (Limayem and Ricke, 2012). After fermentation, lignin residuals and biogas from wastewater treatment can be combusted in an auxiliary boiler to generate electricity for on-site use or exports (Humbird et al., 2011). In terms of CO_2_ capture efficiency, 11–15% of the biomass carbon is released at the fermentation stage with high purity, and is separated from the fuel, while 25–30% of the carbon remains in the end-product (Humbird et al., 2011; IEAGHG, 2014; Seifkar et al., 2015). The advanced CO_2_ capture design (CCS+) integrates amine-based absorption to capture CO_2_ from the combustion of by-products, which provides a higher overall CO_2_ capture rate of 71%.

Out of the five “BECCS” projects in operation today, four are bioethanol plants integrating CO_2_ capture. The Decatur plant in Illinois permanently stores CO_2_ geologically (Gollakota and McDonald 2014), whereas three utilize the CO_2_ for enhanced oil recovery (EOR) – Bonanza and Arkalon plants in Kansas, Husky Energy plant in Canada (Christopher Consoli, 2019). Fermentation produces a high-purity (99%) gaseous stream consisting only of CO_2_, H_2_O, and small amounts of organic and sulfur compounds. Therefore, purification, dehydration, and compression of fermentation streams can be accomplished at a cost lower than $25 tCO_2_^−1^ avoided (Sanchez et al., 2015). Cellulosic ethanol production costs range between $22–30 GJ^−1^ in literature, where cost variations are mainly associated with the level of revenues from co-electricity production and feedstock costs (Hamelinck et al., 2005; Viikari et al., 2012). Similar to other BECCS-to-biofuel pathways, this study adopts the process cost and parameters from the 2020 IEAGHG study on biorefineries with CCS, which facilitates the evaluation of different biofuel pathways on a consistent basis. We consider bioethanol production from corn (EtOH-CCS) as the first-generation feedstock, and miscanthus (CE-CCS/CCS+) as a cellulosic feedstock (Table S1).

##### BECCS for biodiesel (FT-CCS and FT-CCS+)

An alternative BECCS-biofuel pathway is the conversion of cellulosic biomass to fuel, *e.g.*, biodiesel, kerosene, through gasification and Fischer-Tropsch (FT) synthesis. Typical process energy efficiencies reach 42–43%, which includes the energy penalty associated with CO_2_ compression (Liu et al., 2011; Koornneef et al., 2012). Compared to bioethanol production routes, a larger quantity of CO_2_ can be captured during the conversion process, with capture efficiencies ranging from 51 to 56%, while 23–33% of the biomass carbon is found in the FT fuel (Liu et al., 2011; Koornneef et al., 2012). The FT-CCS + route captures additional CO_2_ from flue gas that arises from the combustion of purge from FT synthesis and char from the fluidized-bed gasifier.

FT is considered the most developed and mature technology for synthesis of liquid transportation fuels. Large-scale FT plants worldwide employ either gasification of coal or reforming of natural gas to generate syngas. The adoption of biomass in the process has been explored in various demonstration plants worldwide (Sikarwar et al., 2017) while the inclusion of CCS in the production route has been widely assessed in literature. Liu et al., investigated the techno-economic performance of alternative FT-CCS designs, and found an FT production cost of around 28 $ GJ^−1^, when using switchgrass as a feedstock (Liu et al., 2011). The study also found that for carbon prices higher than 120 $ tCO_2_^−1^, producing FT diesel becomes cheaper than ethanol due to the much higher CO_2_ capture rate of the FT-CCS case (54%–56%) compared to EtOH-CCS (16%).

##### BECCS for hydrogen (H_2_-CCS and H_2_-CCS+)

For hydrogen generation, biomass processing pathways include gasification, pyrolysis, liquefaction and hydrolysis. Gasification has one of the highest stoichiometric yield of hydrogen and is often presented as a promising option based on economic and environmental considerations and is the focus here (Balat and Kırtay, 2010). Biomass gasification is closely related to coal gasification, consisting of steam gasification, gas cleaning (removal of ash and contaminants), water-gas-shift and hydrogen separation via pressure swing adsorption. Gasification with steam reforming of the syngas and water-gas-shift can reach hydrogen yields of 37–50% on an energy basis (Koroneos et al., 2008; IEAGHG, 2014; Parthasarathy and Narayanan, 2014). Absorption separates CO_2_ from the syngas following the water-gas-shift stage and from the auxiliary furnace flue gas that arises from combusting the residue/exhaust gas exiting the hydrogen purification stage (Antonini et al., 2018). The CO_2_ capture efficiency typically reaches 90%. Biomass gasification occurs at temperatures from 500 to 1400°C and operating pressures from atmospheric to 33 bar.

Although biomass gasification for hydrogen production as a whole process is not commercialized for BECCS applications, the individual components are technically mature. Gasification, gas clean up tech, water-gas-shift reactors, CO_2_ absorption, Air Separation Units (ASUs) *etc.,* are commercially available as individual units and used in fossil fuel applications. Like coal gasification, the cost of hydrogen production from biomass are most sensitive to the high cost of capital. Capital costs of solid fuel gasification facilities are expected to reduce with the development of projects at larger scale. Hydrogen production costs are estimated to be $1.82–2.11 kg^−1^ for an output capacity of 139.7 t H_2_ day^−1^ with biomass costs of $47.4–82.5 dry ton^−1^ (Parkinson et al., 2019).

##### BECCS for electricity (Biopower-CCS)

The BECCS pathways predominant in IAMs involve biomass combustion such as a pulverized combustion boiler (PC), or fluidized bed reactor (FBR), and gasification such as integrated gas combined cycle (IGCC) to produce electricity. Co-production of heat is also possible when using a combined heat and power plant (CHP). The CO_2_ capture technology varies as a function of the conversion process: post-combustion capture of CO_2_ (absorption or adsorption), oxy-combustion, and pre-combustion capture (with biomass gasification). The efficiency of BECCS power generation can be as low as 17% in small-scale plants (Hetland et al., 2016) and as high as 37%. Depending on the efficiency of the base plant, process design improvements could potentially increase efficiency up to 38–42% (Koornneef et al., 2012; Bui et al., 2020). Compared to pulverized combustion plants, the efficiency of IGCC plants is typically higher at around 43% with potential improvement to 50% (Koornneef et al., 2012). However, uncertainties remain around the commercialization potential of biomass IGCC, which remains at the pilot scale. Across all technologies, CO_2_ capture efficiencies between 90% and 95% can be achieved (Bui et al., 2018). Advanced absorption technologies can achieve CO_2_ capture rates as high as 99% at a marginal cost increase (Feron et al., 2019; Hirata et al., 2020; Jiang et al., 2020).

The 2.6 GW Drax power plant (UK) was the first to demonstrate CO_2_ capture from biomass flue gas with a pilot-scale absorption-based facility, which captured one tCO_2_/day (Clayton, 2019). The second BECCS demonstration is a facility in Japan, which captures 500 tCO_2_/day from the Mikawa biomass-fired power station. Numerous modeling studies have investigated the techno-economic potential of BECCS deployment in the power sector. The most recent review of CDRs in terms of scale and economics (Fuss et al., 2018) indicate the costs for combustion BECCS in the range of 88–288 $/tCO_2_, with wide variations can be attributed to differences in modeling assumptions and boundaries conditions. In this study, we adopted the MONET database (Fajardy et al., 2018) to derive the capital and operational costs associated with the deployment and operation of a reference 500 MW BECCS plant in Europe. The MONET database uses background data from the World Energy Outlook (International Energy Agency (IEA), 2016).

##### Biomass in iron and steel (BECCS-BF and BECCS-DRI)

Biomass-based raw materials can also be utilized in other industrial processes, such as in the petrochemical and iron and steel industries. In steelmaking processes that employ the blast furnace and basic oxygen furnace (BF-BOF), biomass products in the form of charcoal can substitute pulverized coal in the blast furnace (BF), or partially replace coke and sinter (Mousa et al., 2016; Mandova et al., 2018). For steelmaking processes using DRI, wood-based syngas can replace natural gas as the reducing agent in the DRI furnace; this has already been demonstrated with syngas derived from coal. In both steelmaking routes, the biomass products provide the thermal energy required for the production of liquid steel. To ensure the permanent removal of biomass carbon, the steelmaking plant would need to be coupled with post-combustion capture to separate the CO_2_ from the furnace off-gases, which represent approximately 60% of the plant's CO_2_ emissions. Alternatively, CO_2_ may be captured from the various other carbon sources, *i.e.*, the pellet, coke and sinter plants, however, this would only provide small marginal CO_2_ abatement benefits (Tanzer et al., 2020) at potentially significant marginal cost, thus direct abatement may not prove to be cost-optimal in this case.

At the time of writing, the use of BECCS in the steel industry has yet to be demonstrated in practice. A recent study (Tanzer et al., 2020) estimated the CO_2_ balance associated with BECCS integration in multiple steelmaking routes, including BF and DRI. Process modeling and life cycle assessment were used to estimate life cycle emissions at process level. Here, we build on the findings obtained from that study and enhance the analysis with the calculation of the cost and economics associated with the integration of BECCS in both BF and DRI.

#### Techno-economic assessment

For each of the conversion routes presented in Figure 1, our analysis considers the main costs occurring at various stages of the biomass value chain, including feedstock cultivation and transport, operational and capital expenditure of a refence plant, and CO_2_ transport and storage. Table S1 present the main techno-economic parameters for the reference biomass conversion pathways adopted in the analysis, Table S2 summarizes the results of the economic analysis at technology level.

Some biomass pathways, involve the co-production of electricity as indicated by the Net Electricity balance in Table S1, these are:

• BECCS to bioethanol pathways, involving the production of electricity from the combustion of residual lignin in bioethanol plants;
• Biochar production *via* pyrolysis, which yield different proportions of biochar and syngas, which is then converted to electricity.

Thus, our analysis considers potential revenues generated by exploiting surplus of electricity to the grid, considering the average European electricity market price in 2019.

#### Carbon avoided and removed

The estimation of the carbon removal and avoidance potential of the proposed biomass pathways, is associated with the following key parameters, summarized in Table S3:

• Biomass energy density and carbon content;
• Biomass carbon footprint, which includes CO_2_ emissions associated with biomass production, processing (*e.g.*, drying) and biomass transport;
• Supply chain dry mass recovery, which captures biomass dry mass loss along the value chain;
• Process capture efficiency, which characterizes the amount of CO_2_ captured from the biomass conversion process;
• Post capture efficiency, which accounts for potential CO_2_ leakage associated with downstream energy use, *i.e.*, CO_2_ transport and injection for BECCS pathways, biochar distribution;
• Storage efficiency, which measures the amount of CO_2_ buried/injected and effectively stored;
• The process energy efficiency, which measures the conversion efficiency of biomass to energy.

For each pathway, two metrics are calculated: net CO_2_ removal per unit of biomass carbon, and CO_2_ avoided per unit of biomass carbon, C_bio_.

For each pathway, *p*, and biomass, *b*, net CO_2_ removal is calculated as the physical amount of biogenic CO_2_ fixed in geological or natural storage, to which the life cycle fossil emissions of this pathway (*e.g*., biomass production) are subtracted:

$$Net\;CO_{2}\;removal\;\left(b,p\right)=\;\frac{C_{bio}\left(b\right)\frac{44}{12}.SR\left(b\right).\eta_{C}\left(p\right).\eta_{PC}\left(p\right).\eta_{S}\left(p\right)-CF_{bio}\left(b\right)}{C_{bio}\left(b\right)}\;$$

The amount of CO_2_ avoided is somewhat more complex to quantify as it is entirely dependent on the counterfactual, *c*, chosen for each scenario. When possible, high, average, and low carbon intensity counterfactuals were chosen to determine a CO_2_ avoidance range. Depending on the pathway, different sources of CO_2_ avoidance were considered:

• When low carbon energy is generated and displaces another form of energy generation, with a substitution factor, considering the process efficiency of the pathway, *η*_*E*_*(p)*, the energy substitution factor, *EF*_*E*_*(p)*:

$$CO_{2}\;avoided,\;E\left(b,p,b\right)=\;\frac{SR\left(b\right).\;HHV_{bio}\left(b\right).\eta_{E}\left(p\right).Sub_{E}\left(c\right).EF_{E}\left(p\right)}{C_{bio}\left(b\right)}$$

• When the low carbon product (timber and biochar), displaces other carbon intensive products with a product substitution factor, EF_P_(p):

$$CO_{2}\;avoided,\;P\left(b,p,c\right)=\;\frac{SR\left(b\right).\;\eta_{P}\left(p\right).Sub_{p}\left(c\right).EF_{P}\left(c\right)}{C_{bio}\left(b\right)}$$

While life cycle CO_2_ emissions are usually considered in the calculation of avoided CO_2_ emissions, the rationale for including them in the net CO_2_ removal is that these solely depend on the pathway p, independent of any counterfactual scenario considered for the quantification of avoided CO_2_ emissions. Table S4 summarizes the counterfactuals considered for each pathway and the associated assumptions.

## Supporting citations

The following references appear in the supplemental information (Berkenpas, 2001; Gaunt and Lehmann, 2008; Vassilev et al., 2010; Woolf et al., 2010; Liu et al., 2011; Shackley et al., 2011; Humbird et al., 2011; Nhuchhen and Abdul Salam, 2012; Smith and Torn, 2013; IEAGHG, 2014; Skullestad et al., 2016; Sustainable Gas Institute, 2017; Fajardy and Mac Dowell, 2017; Antonini et al., 2018; Fajardy et al., 2018; IEA, 2019; Tanzer et al., 2020; Das et al., 2020; Forest Research, 2020; Hannula and Melin, 2021):

## Footnotes

Supplemental information can be found online at https://doi.org/10.1016/j.isci.2021.102765.
