# Supplementary material for: CO2 mitigation or removal: The optimal uses of biomass in energy system decarbonization
Source: iScience. 2021 Jun 24;24(7):102765. doi: 10.1016/j.isci.2021.102765 (PMC8283136; doi:10.1016/j.isci.2021.102765)
Supplement: Document S1. Tables S1–S5 [file mmc1.pdf]

iScience, Volume *24*

Supplemental information

CO_2 mitigation or removal: The optimal uses of biomass in energy system decarbonization

Piera Patrizio, Mathilde Fajardy, Mai Bui, and Niall Mac Dowell

CO_2 mitigation or removal: the optimal uses of biomass in energy systems decarbonization

Supplementary information

1. Selected biomass-based CO_2 removal pathways: process performance and costs

Main product	Reference unit (RU)	Main source	Pathway	Plant odt (Mtdry/yr)	Net electricity balance (GJ/RU)	CO_2 captured (kg_{co2}/RU)
Timber	m^3 CLT	(Brandt et al. 2019)	CLT	0.02	N/A	772
Biochar	t biochar	(Shackley et al. 2011)	Slow pyrolysis	0.18	10.91	2178
Bioethanol	GJ fuel	IEAGHG (2020)	EtOH CCS	0.89	-0.13	46.7
			CE-CCS/CCS+	0.38	0.45/0.61	37.3/275.7
FT diesel			FT-CCS/CCS+	0.2	0.01/0.03	122/150
Biohydrogen			H_2-CCS/CCS+	0.15	-0.36/ -0.37	156/167
Bioelectricity	kWh	Fajardy (2018)	Biopower CCS	2.75	N/A	427
Bio-steel	ton HRC	Tanzer et al (2020), IEAGHG (2013)	BECCS-DRI	1.59	N/A	1100
		Tanzer et al (2020), Lockwood (2000)	BECCS-BF	1.85	N/A	900

Table S1: Cost breakdown for the selected biomass conversion pathways, related to STAR Methods. Note that in the case of BECCS in iron and steel, these are brownfield investments, hence the total production costs represent marginal cost increases of retrofitting existing steel mills with BECCS

Pathway	RU	Feedstock cost [(a)]	CAPEX [(b)]	OPEX	Revenues from electricity production [(c)]	Downstream costs [(d)]	Total production costs
CLT	$\$_{2018}/m^3$ CLT	65.78	171	411	0	N/A	647.8
Slow pyrolysis	$\$_{2018}$/t biochar	150	110	14	-145	10	139
EtOH CCS	$\$_{2018}$/GJ	16.15	2.2	0.9	0.77	1.4	21.4
CE CCS		21.34	15.2	6	0	1.12	43.7
CE CCS+		21.34	15.3	8.1	0	8.27	53
FT CCS		11.17	24.2	10.3	0	3.66	49.3
FT CCS+		11.17	27.3	10.7	0	4.5	53.7
H2-CCS		8.56	26.2	10.3	1.81	4.68	51.6
H2-CCS+		8.56	26.8	10.5	1.88	5.01	52.8
Biopower CCS		26.6	33.23	10.75	0	12.81	83.4
BECCS-DRI	$\$/t_{HRC}$	109.9	74	12	0	33	228.9
BECCS-BF		25.9	53.5	20.02	0	27	126.4

[(a)] The following feedstock costs have been assumed: wood chips: 70 $/ton (Fajardy and Mac Dowell, 2017), corn: 118 $/ton (Hannula and Melin, 2020), miscanthus pellets: 96 $/ton, timber: 50 $/ton (Forest Research, 2020), weath straw: 51 $/ton (Fajardy and Mac Dowell, 2017).

[(b)] We calculate the Total Capital Investment (TCI) based annual capital charges using 0.12 capital charge rate, which is based on 10% WACC (weighted average cost of capital) and 20-year economic life for the biomass conversion plants. Value are represented in $ 2018

[(c)] Our analysis considers potential revenues generated by exploiting surplus of electricity to the grid, considering an average electricity selling price of $49 MWh^{-1} for 2019 based on the European Network of Transmission Systems Operators (ENTSO).

[(d)] The cost associated with the downstream activities such as biochar spreading in the field, (Shackley *et al.*, 2011), and CO_2 transport and storage, assuming an average value of 30 $\$/t_{CO2}$

Table S2: Cost breakdown for the selected biomass conversion pathways, related to the STAR Methods. Note that in the case of BECCS in iron and steel, these are brownfield investments, hence the total production costs represent marginal cost increases of retrofitting existing steel mills with BECCS

Pathway	Main product	Biomass	Biomass HHV (GJ/t_{odt})[a] HHV_{bio}	Biomass Carbon Content[a] (%$_{odt}$) C_{bio}	Biomass carbon footprint[b] (kgCO_2/t_{odt}) CF_{bio}	Supply chain dry mass recovery[c] SR	Process capture efficiency (%$_{CO2}$)[d] η_C	Post capture efficiency (%$_{CO2}$)[e] η_{PC}	Storage efficiency[f] (%$_{CO2}$) η_S	Process energy efficiency[g] (%$_{LHV}$) η_E
Cross Laminated Timber	Timber	Timber	19.5	51.6	213	90%	N/A	N/A	100%	N/A
Slow pyrolysis	Biochar/electricity	Residues (wheat straw)	18	45.9	67	95%	49%	96%	68%	13%
EtOH-CCS	Bioethanol	Corn	16.6	44	273	95%	21%	94%	100%	44%
CE-CCS/CCS+	Bioethanol		19.2	47.7	176	95%	14%/71%	94%	100%	23%
FT-CCS/ CCS+	Ft diesel	Miscanthus	19.2	47.7	176	95%	54/66%	94%	100%	43%
H2-CCS/ CCS+	Biohydrogen		19.2	47.7	176	95%	89%/96%	94%	100%	37%
Biopower CCS	Bioelectricty	Pellet (miscanthus)	19.2	47.7	176	90%	95%	94%	100%	26%
BECCS-DRI	Bio-steel	wood chips	19.5	51.5	37.8	90%	90%	94%	100%	33%
BECCS-BF	Bio—steel	Wood chips	19.5	51.5	37.8	90%	63%	94%	100%	66%

(a) Biomass carbon content are from Vassilev et al. (Vassilev *et al.*, 2010) and higher heating values from Nhuchhen and Abdul Salam (2012) (Nhuchhen and Abdul Salam, 2012).

(b) The carbon footprint of biomass pellets is an European average calculated in the MONET framework (Fajardy, Chiquier and Mac Dowell, 2018) and includes biomass production (seed, fuel for land preparation and harvest, fertiliser direct and indirect $CO_{2,eq}$ emissions), pelletising, average distance transport (100-200km), and pellet grinding. The carbon footprint of agricultural residues is also an European average calculated in MONET and include straw collection, additional fertiliser application to compensate for straw removal, drying, chopping and 50k transport.

c) It is assumed biomass dry mass is lost during transport and processing, at the rate of 5% for residues (drying, local transport), and 10% for timber (drying, >100km transport) and pellets (pelletising, >100km transport).

(d) It is assumed that biomass initial carbon content is captured at 100% in timber, 49% in biochar (Woolf et al. 2010), 14% from the biomass fermentation to ethanol (Humbird *et al.*, 2011), 56% from biomass gasification for biodiesel (Liu *et al.*, 2011), 90% for biomass gasification to hydrogen (IEAGHG, 2014; Antonini *et al.*, 2018), 95% for biomass combustion to electricity. For biomass in iron and steel industry, 63% of initial biomass carbon is captured in BF-BOF route and 34% in the case of DRI-EAF (Tanzer, Blok and Ramírez, 2020)

(e) This parameter captures any post conversion process emissions associated with energy use including 4% CO_2 emissions tilling and spreading for biochar (Gaunt and Lehmann 2008), 6% emissions associated with CO_2 transport and storage (Smith and Torn, 2013).

(f) Out of the CO_2 sequestered in timber and geological storage, 100% of the CO_2 is considered fixed and stored. For the biochar process, it is assumed that 68% of the biochar carbon is fixed in the soil (Gaunt and Lehmann, 2008)

(g) Process efficiency of biomass conversion (in electricity or fuel LHV/biomass HHV) is considered to be 13% for biomass to bioelectricity via pyrolysis (Gaunt and Lehmann, 2008), 44% for biomass to bioethanol (Humbird *et al.*, 2011) 43% for biomass to biodiesel (Liu *et al.*, 2011), 37% for biomass to hydrogen (IEAGHG, 2014), and 26% for biomass to electricity (simulation in IECM see (Berkenpas *et al.*, 2001)).

Table S3: Summary of key parameters and assumptions adopted in the carbon balance of each biomass pathways, related to the STAR Methods.

<table>
<tr><th>Pathway</th><th>Counterfactual(s)</th><th>Energy substitution factor[a]
Sub_E</th><th>EF of energy displaced[b] (gCO_2/MJ)
EF_E</th><th>Useful product (ton/odt)
η_p</th><th>Product substitution factor
Sub_P</th><th>CF of product displaced ($kgCO_2/kg$)[b]
EF_P</th></tr>
<tr><td>Timber[c]</td><td>Concrete</td><td>N/A</td><td>N/A</td><td>100%</td><td>29%</td><td>189</td></tr>
<tr><td rowspan="3">Biochar and bioelectricity[d]</td><td>a1) Fertiliser use
a2) High carbon intensity electricity grid (e.g., Poland)</td><td>100%</td><td>224</td><td>49% of C_{bio}</td><td rowspan="3">Biochar application rate 5 t_C/ha
10% less fertiliser needed
50% less N_2O emissions from fertiliser</td><td rowspan="3">3.44 $kgCO_{2,eq}$/kg N
1.25% kg N_2O/kgN
GWP(N2O) = 265</td></tr>
<tr><td>b1) Fertiliser use
b2) Average carbon intensity electricity grid (World)</td><td>100%</td><td>51</td><td></td></tr>
<tr><td>c1) Fertiliser
c2) Low carbon intensity electricity grid (e.g., Sweden)</td><td>100%</td><td>6</td><td></td></tr>
<tr><td rowspan="2">Bioethanol CCS</td><td>Gasoline</td><td>100%</td><td>69</td><td rowspan="9" colspan="3">N/A</td></tr>
<tr><td>EV (in low carbon grid)[e]</td><td>26%</td><td>3.7</td></tr>
<tr><td rowspan="2">Biodiesel CCS</td><td>Gasoline</td><td>100%</td><td>69</td></tr>
<tr><td>EV (in low carbon grid)</td><td>100%</td><td>3.7</td></tr>
<tr><td rowspan="2">Biohydrogen CCS</td><td>Natural gas</td><td>100%</td><td>55</td></tr>
<tr><td>Electrolyser with wind H_2</td><td>100%</td><td>6.9</td></tr>
<tr><td rowspan="3">Bioelectricity CCS</td><td>High carbon intensity electricity grid (e.g., Poland)</td><td>100%</td><td>215</td></tr>
<tr><td>Average carbon intensity electricity grid (Europe)</td><td>100%</td><td>64.1</td></tr>
<tr><td>Low carbon intensity electricity grid (e.g., Sweden)</td><td>100%</td><td>3.7</td></tr>
<tr><td colspan="7">[a] Bioethanol and biodiesel were assumed to have the same fuel economy in MJ/km than conventional gasoline or diesel. [b] Average carbon intensity of electricity from 2016 was collected from the European Environmental Agency (EEA) database . The gasoline and nitrogen fertiliser emissions factors used in the MONET framework were used here for consistency with biomass carbon footprint values. The carbon intensity of hydrogen generated by natural reforming with CCS and wind-powered electrolyser was obtained from (Sustainable Gas Institute, 2017). [c]Data relative to biochar carbon yield, application rate and impact on fertiliser-related emissions were taken from (Gaunt and Lehmann, 2008). [d]Data related to timber/concrete substitution and avoided emissions were taken from (Skullestad, Bohne and Lohne, 2016). [e] Calculated considering the average electricity consumption of an EV of 166 Wh/km (Das et al., 2020) and the 2017 world average of gasoline energy consumption for internal combustion engines of 2.30 MJ/km (IEA 2019).</td></tr>
</table>

Table S*4*: Counterfactual specific assumptions, related to Figure 2.

2. Fuel emissions and costs

Fuel	Emission factor ($kgCO_2/MWh$)	Market price ($/GJ)
Electricity (Germany) [a]	440.8	10.65
Electricity (Poland)	773.3	14.2
Electricity (Sweden)	13.3	11.05
Electricity (EU28)	291.3	11.4
Natural gas (Germany)	208.4	17.55
Natural gas (Poland)		13.13
Natural gas (Sweden)		32.86
Natural gas (EU28)		16.61
Gasoline	89.7	18.1
[a]Hourly day ahead prices were used from ENTSOE and the average was taken for each country in 2019. [b] Natural gas prices are derived from EUROSTAT website		

Table S5. Techno-economic parameters of main fuels adopted in the counterfactual scenarios, related to the STAR Methods.
